# Efficient Computational Modeling of Human Ventricular Activation and Its Electrocardiographic Representation: A Sensitivity Study

**DOI:** 10.1007/s13239-018-0347-0

**Published:** 2018-03-16

**Authors:** Jonathan P. Cranford, Thomas J. O’Hara, Christopher T. Villongco, Omar M. Hafez, Robert C. Blake, Joseph Loscalzo, Jean-Luc Fattebert, David F. Richards, Xiaohua Zhang, James N. Glosli, Andrew D. McCulloch, David E. Krummen, Felice C. Lightstone, Sergio E. Wong

**Affiliations:** 1 Lawrence Livermore National Laboratory, 7000 East Avenue L-126, Livermore, CA 94550 USA; 2 University of California, San Diego, 9500 Gilman Dr., La Jolla, CA 92093 USA; 3 University of California, Davis, 1 Shields Ave, Davis, CA 95616 USA; 4 Harvard Medical School, 25 Shattuck St., Boston, MA 02115 USA; 5 Brigham and Women’s Hospital, 75 Francis Street, Boston, MA 02115 USA; 6 VA San Diego Healthcare System, 3350 La Jolla Village Dr., San Diego, CA 92161 USA

**Keywords:** Human ventricular excitation, Sensitivity analysis, Electrocardiogram, Patient-specific modeling, Computational electrophysiology, Bundle branch block

## Abstract

Patient-specific models of the ventricular myocardium, combined with the computational power to run rapid simulations, are approaching the level where they could be used for personalized cardiovascular medicine. A major remaining challenge is determining model parameters from available patient data, especially for models of the Purkinje-myocardial junctions (PMJs): the sites of initial ventricular electrical activation. There are no non-invasive methods for localizing PMJs in patients, and the relationship between the standard clinical ECG and PMJ model parameters is underexplored. Thus, this study aimed to determine the sensitivity of the QRS complex of the ECG to the anatomical location and regional number of PMJs. The QRS complex was simulated using an image-based human torso and biventricular model, and cardiac electrophysiology was simulated using Cardioid. The PMJs were modeled as discrete current injection stimuli, and the location and number of stimuli were varied within initial activation regions based on published experiments. Results indicate that the QRS complex features were most sensitive to the presence or absence of four “seed” stimuli, and adjusting locations of nearby “regional” stimuli provided finer tuning. Decreasing number of regional stimuli by an order of magnitude resulted in virtually no change in the QRS complex. Thus, a minimal 12-stimuli configuration was identified that resulted in physiological excitation, defined by QRS complex feature metrics and ventricular excitation pattern. Overall, the sensitivity results suggest that parameterizing PMJ location, rather than number, be given significantly higher priority in future studies creating personalized ventricular models from patient-derived ECGs.

## Introduction

As modeling and computational methodologies evolve, the ability to use these techniques to provide more effective, customized medical care for patients is fast approaching, generally termed “patient-specific modeling”.^1^,^28^,^32^ Even now, the potential applications of patient-specific modeling has drawn attention from NIH in the form of numerous funding announcements.^15^ Patient-specific modeling facilitates the ability to move away from using population-based metrics to prescribe treatment for an individual, a practice that does not result in optimal care for many patients.^30^ It has the potential to reduce dependence on “trial and error” techniques to determine a patient’s response to a particular treatment, and to move towards lower risk, more effective, truly personalized therapy.^30^

Despite the promise and excitement of the clinical implications of patient-specific modeling, it has not yet advanced to the point where it can be used as a standard of clinical care. One of the ongoing challenges is determining model parameters from available patient data in a minimally invasive fashion.^10^ In many cases, parameters of larger scale geometries may be obtained, yet parameters related to detailed structures are more difficult to obtain. This challenge is particularly evident in modeling initial electrical activation of the ventricles. Initial ventricular activation is crucial for coordinated ventricular contraction, and activation disorders include bundle branch block (BBB), higher-degree heart block, and symptomatic profound sinus bradycardia. Yet, the Purkinje network that carries the electrical signal to the ventricular muscle is too fine to be captured by *current clinical* imaging technologies, and the Purkinje-myocardial junctions (PMJs) that activate the muscle are even more elusive.^17^,^31^

Previous studies that have modeled initial ventricular activation have offered valuable insights into obtaining appropriate model parameters; yet, there are still challenges in implementing them in the context of patient-specific modeling. Some approaches yield very detailed and specific information, but require extracting the heart and imaging intact or dissected ventricles,^5^,^6^,^11^,^23^,^24^,^27^ which is not viable for designing customized treatment of patients in the clinic. Other approaches use a fractal tree rule-based approach for growing the Purkinje network and defining the PMJs along the subendocardium, which enables parameter exploration and sensitivity analyses of model parameters.^3^,^8^,^17^,^31^ Some of these rule-based approaches use activation times from patient endocardial mappings to customize the network and junctions to a particular patient^17^,^31^; yet, this requires an invasive procedure that still does not guarantee exact architecture of the patient’s Purkinje network. Furthermore, while full representation of the Purkinje network and PMJs may be required for certain disorders of initial activation, customizing this full network drastically increases the parameter space that must be explored in parameter fitting. Finally, a limited number of studies are emerging that explore the relationship between PMJ model parameters and the QRS complex of the standard, noninvasive ECG.^7^,^19^ These studies offer initial insight into using patient-derived ECGs to determine PMJ model parameters, yet only a subset of the possible parameters and their interactions have been fully explored.

Our work begins by recognizing the potential benefit of determining PMJ model parameters from the patient-derived, noninvasive QRS complex of the ECG that is commonly measured in the clinic. We posit that an important step in advancing this approach is to further determine the sensitivity of the QRS complex to properties of the PMJs, and in turn, which PMJ properties are most important in model parameter fitting. To perform this analysis, we build upon previous work by our team, having already successfully demonstrated a simulation of several heart beats and the resulting QRS complexes in an image-based biventricular and torso model.^12^,^20^ Specifically, we use our existing codes and tools to explore the sensitivity of the simulated QRS complex to ventricular model input parameters related to regional number and anatomical location of PMJs, modeling the PMJs as discrete current-injection activation stimuli. Number and location of PMJs are important parameters that have been explored previously,^3^,^14^ but their interplay and relative importance have not been fully explored in the context of the QRS complex.^7^,^19^ As an extension to our sensitivity analysis in healthy ventricles, we also simulate the QRS complex features seen in LBBB and RBBB. Finally, as a control and to provide greater connection to other models in the community that calculate the QRS complex in different ways, we repeat the sensitivity analysis using an alternate model for calculation of the QRS complex.

## Materials and Methods

### Biventricular and Torso Models Used in Sensitivity Analysis

Simulations of ventricles were executed on the IBM Blue Gene^®^/Q supercomputers at Lawrence Livermore National Laboratory (LLNL), using the highly scalable code Cardioid. Cardioid solves the reaction-diffusion equations of the monodomain model, which describes spatiotemporal evolution of the transmembrane potential (*V*_m_),

1 $$C_{\text{m}} \frac{{\partial V_{\text{m}} }}{\partial t} = \frac{1}{\beta }\nabla \cdot (D\nabla V_{\text{m}} ) - I_{\text{ion}} + I_{\text{stim}} ,$$

where *Cm* is the membrane surface capacitance, *t* is time, *β* is the tissue surface area-to-volume ratio, *D* is the spatially-dependent anisotropic conductivity tensor determined by the fiber structure of the heart, and *I*_stim_ is the imposed stimulus. The reaction term, *I*_ion_, represents the sum of the nonlinear ionic current densities at the cellular level, using the 2006 ten Tusscher model^26^ with modifications as described in our previous publications.^12^,^20^

Equation (1) is solved on the domain comprised of geometry from images of human ventricles obtained from the Visible Human Project^®^ (VHP) of The National Library of Medicine.^29^ To obtain this ventricular domain, the full torso from VHP was segmented and meshed as follows: two-dimensional full-body cryosection images were stacked together to form a three-dimensional image. The 3D image was segmented using a combination of thresholding, level set, and manual techniques using the software package Seg3D.^21^ Next, a linear tetrahedral finite element mesh was generated from the segmented image. The resulting mesh contained 11 different tissues in the torso and was conformal along interfaces, including multiple-material interfaces. The ventricular mesh was extracted from the full torso mesh, and used to generate a 3D Cartesian grid (0.1 mm resolution), which is the domain in which Eq. (1) is solved using finite volumes for the divergence operator and finite differences for the gradient operator. Muscle fiber orientations were generated using a rule-based approach similar to that of Bayer *et al.*^2^ A forward Euler scheme is used for time integration. For additional detailed information, see prior Cardioid publications.^12^,^20^

**Figure 1:**
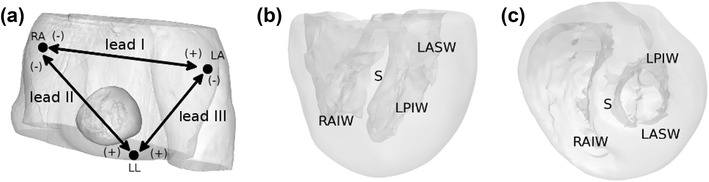
Transverse cross-section of the torso and heart mesh used to compute the ECG leads (a), and four IARs in the biventricular model based on experiments by Durrer *et al.*^9^ (b and c). In (a), locations of left arm (LA), right arm (RA), and left leg (LL) electrodes are indicated by black circles, and leads I, II, and III are labeled according to the potential differences indicated by the arrows. Internal torso tissues exterior to the heart (e.g., bone and fat) are not shown for clarity. The four IARs in the ventricles are shown from the anterior view (b) and from the superior view (c); acronyms are expanded in the text.

The combined torso and ventricular models together enabled computation of the QRS complex of the ECG, as described previously.^20^ Note, only the QRS complex was computed, not the T wave, given that our study is focused entirely on ventricular activation. Surface potential was calculated on the left arm, right arm, and bottom left side of the torso, and from differences in these potentials, leads I, II, and III of the ECG were derived (Fig. 1a). The sensitivity of four QRS complex features were analyzed: Q, R, and S wave amplitudes, and the QRS duration. Amplitudes were calculated using the Pan-Tompkins algorithm^18^ in the ECG leads where they were most pronounced based on the heart’s orientation in the model: lead I for Q and R waves, and lead II for the S wave. QRS duration was measured from lead I. Lead III of the QRS complex was also calculated to provide more information about the orientation of the heart in the torso. Simulations of the combined ventricular and torso models provide connection between ventricular level activation parameters and the features of the QRS complex, which ultimately enable the sensitivity analysis.

### Ventricular Stimuli Parameters in the Sensitivity Analysis

Prior to performing sensitivity analysis simulations, four initial activation regions (IARs) in the ventricles were defined based on experiments by Durrer *et al.*^9^ Durrer *et al.*^9^ performed seminal studies nearly half a century ago, which still represent the “gold standard” dataset and remain in wide use.^4^,^8^,^11^,^22^ In their work, activation timings (ATs) were recorded from seven healthy human hearts, each investigated using the Langendorff preparation with 870 intramural plunge electrode recordings. Across all seven hearts, four IARs were found (Figs. 1b and 1c): 1) the left side of the midseptum (S), 2) the left anterior superior wall near the base (LASW), 3) the left posterior inferior wall (LPIW) in the paraseptal region approximately one-third the distance between the apex and base, and 4) the right anterior inferior wall (RAIW) near the insertion of the anterior papillary muscle. The sensitivity analysis was built upon different configurations of stimuli in these IARs. Stimuli were placed subendocardially, where the PMJs reside, to excite the ventricles. Each stimulus was defined as an 8 mm^3^ volume (rectangular parallelepiped) of tissue with an injected stimulus current (*I*_stim_ in Eq. (1)). All stimuli had the following square wave pulse parameters: magnitude = 72 *µ*A/*µ*F, duration = 1.0 ms, period = 1000 ms. All stimuli were initiated at the beginning of the period, except that stimuli in the right ventricular wall were delayed by 5 ms, based on experimental observations of Durrer *et al.*^9^

## Results

### Sensitivity of QRS Complex Features to Number of Seed Stimuli

**Figure 2:**
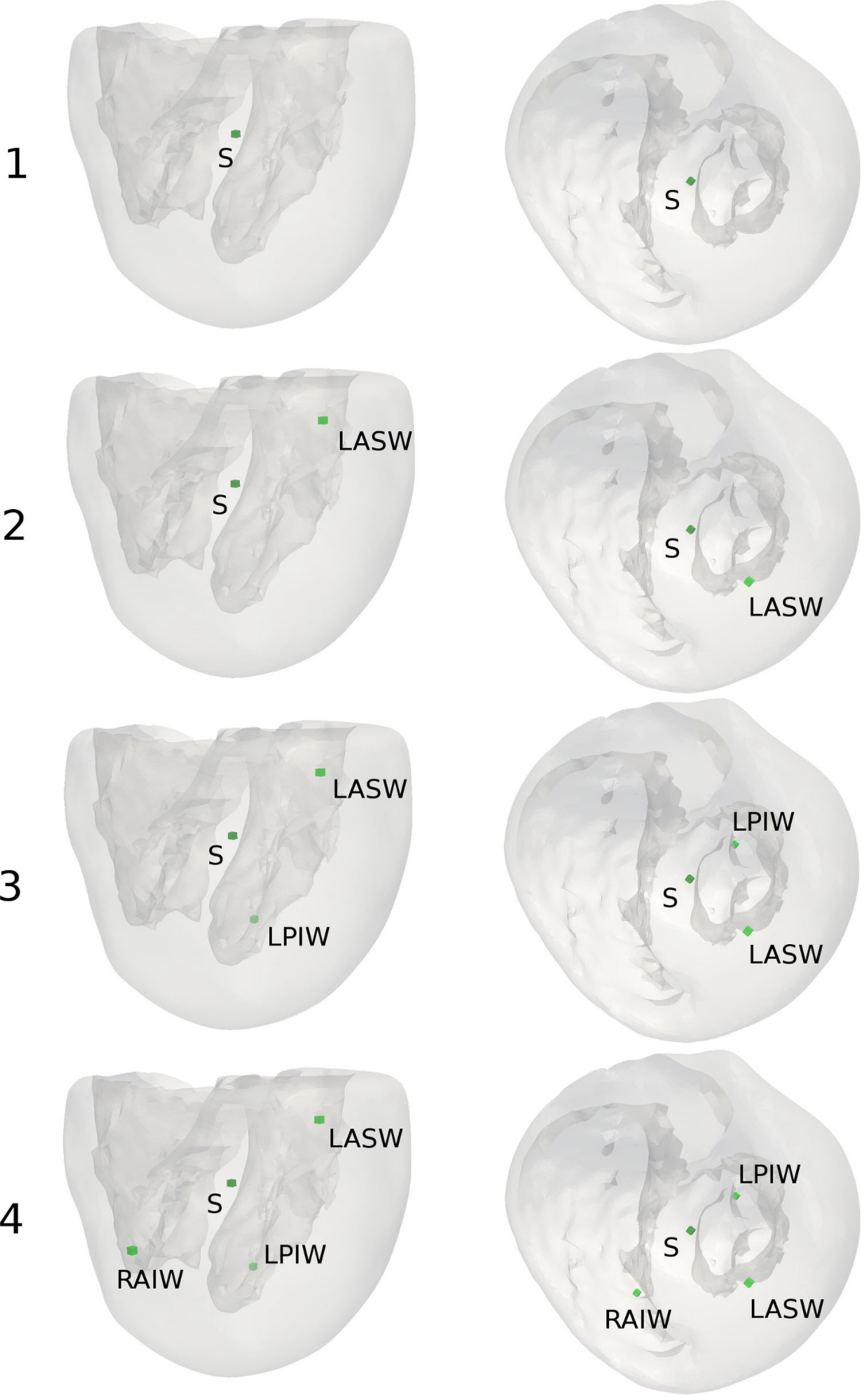
Seed stimuli in the ventricles. The four stimuli are given codes corresponding to the IAR in which they reside (see Fig. 1): septum (S), left ventricular anterior superior wall (LASW), left ventricular posterior inferior wall (LPIW), and right ventricular anterior inferior wall (RAIW). Stimuli are displayed in the order in which they were added in the sensitivity analysis: S, S/LASW, S/LASW/LPIW, and S/LASW/LPIW/RAIW. The anterior view along with the total number of stimuli for each configuration are shown on the left, and the superior view is shown on the right.

**Figure 3:**
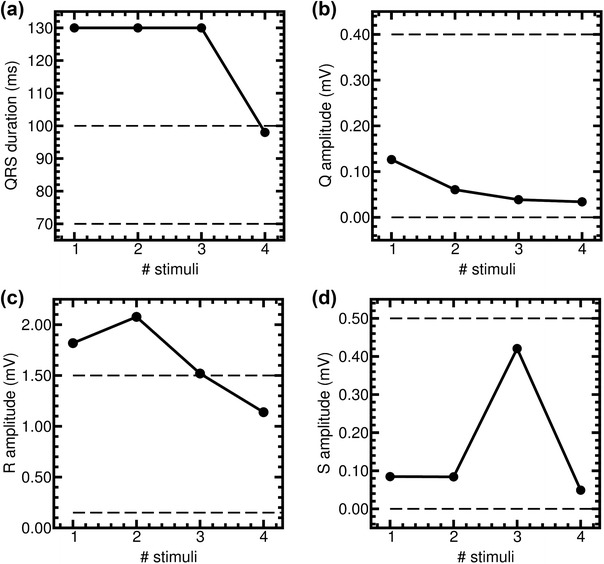
Values of QRS complex features as a function of the total number of seed stimuli, up to a maximum of four stimuli. Stimuli are added in the order presented in Fig. 2. For each panel, the thin dashed lines indicate upper and lower physiological thresholds for the normal QRS complex (from Chou’s Electrocardiography in Clinical Practice^25^). Panels show the QRS duration measured in lead I (a), the Q wave amplitude measured in lead I (b), the R wave amplitude measured in lead I (c), and the S wave amplitude measured in lead II (d). Lead positions on the torso are shown in Fig. 1a.

Analysis begins by investigating the sensitivity of the QRS complex features to number of “seed” activation stimuli. A seed stimulus is a single, initial stimulus for each IAR. One by one, to observe the effect each seed stimulus has on the QRS complex features, each IAR receives a seed stimulus (Fig. 2). Figure 3 presents the QRS complex feature metrics as the number of seed stimuli are increased. Overall, the feature metrics are sensitive to the number of seed stimuli, especially the QRS duration and R wave amplitude, where changing number of seed stimuli by just one can bring these metrics in and out of physiological range (Figs. 3a and 3c). For example, simply adding a stimulus in the right ventricular myocardium changes the QRS duration by nearly 25% and moves it within physiological range. The ranges of QRS durations and R wave amplitudes data are 107% and 70% of their physiological ranges, respectively. In contrast, the Q and S wave amplitudes, while showing some sensitivity, are always within physiological range, even though S wave amplitude varies more significantly (range of data points is 74% of physiological range) than the Q wave amplitude (range of data points is 23% of physiological range). This seemingly anomalous behavior is discussed below in the observed trends.

**Figure 4:**
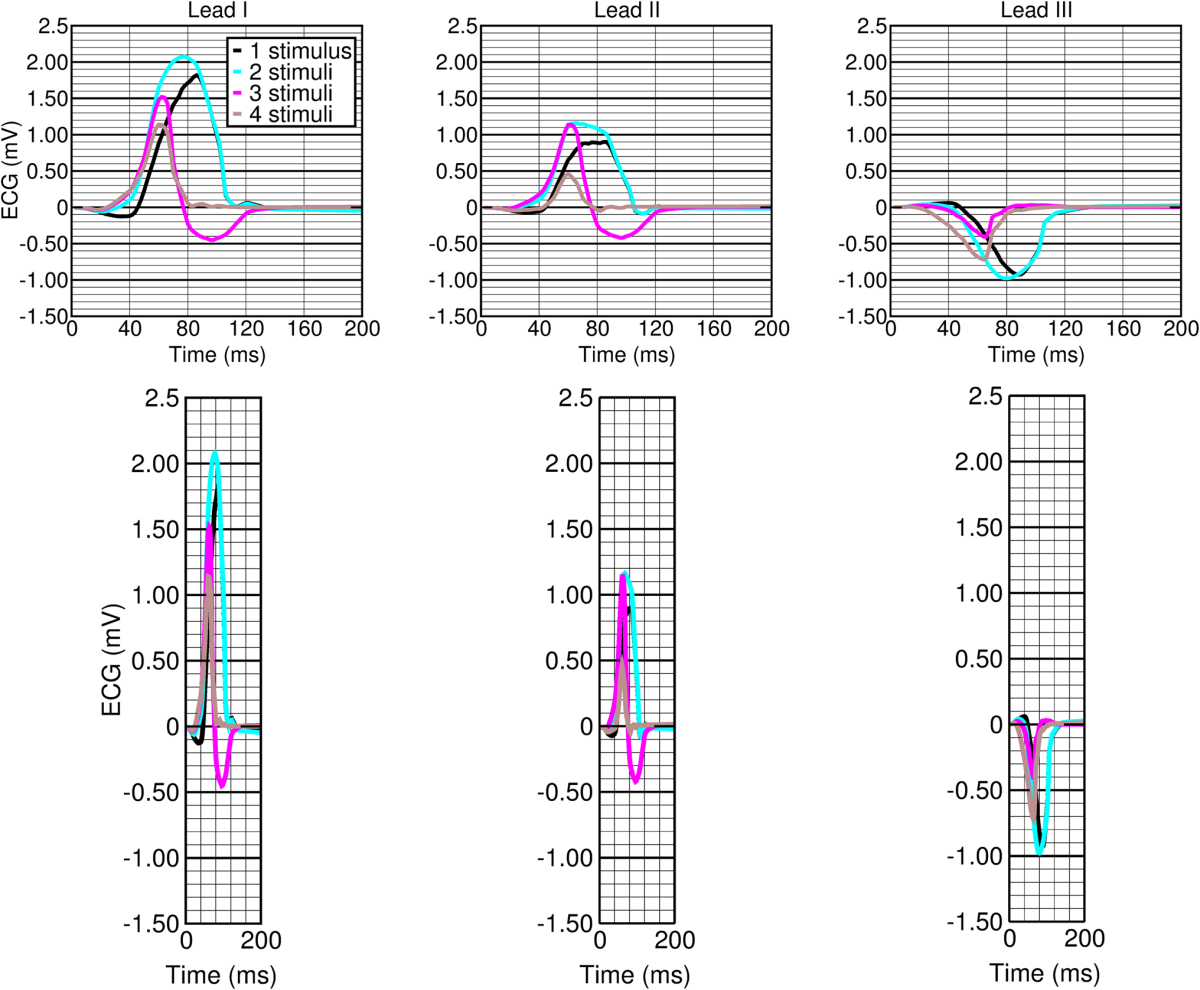
QRS complex of the ECG in leads I–III (lead positions on the torso shown in Fig. 1) as seed stimuli are added, up to a maximum of four total stimuli. Stimuli are added in the order given in Fig. 2. Thick grid lines indicate 0.5 mV and 200 ms increments and thin grid lines indicate 0.1 mV and 40 ms increments, in accordance with the standard ECG. Top row uses x/y-axes ratio not in accordance with the standard ECG to increased clarity, while the bottom row does use the proper ratio where 0.1 mV and 40 ms intervals are equivalent Euclidean distances. Signals scaled to the standard ECG are shown here, for reference, and are generally not shown in later results (non-scaled version is clearer).

The trends observed in the feature metrics as more stimuli are added are not consistent between all metrics. The QRS duration and Q wave amplitude show no change or monotonic decrease as seed stimuli are added, but the R and S wave amplitudes change in a more complex way. Considering each metric in turn, the ultimate decrease in QRS duration is consistent with the understanding that adding stimuli increases the volume of tissue that is activated early, thus decreasing total activation time. The Q wave amplitude decreases monotonically, perhaps from activation of more substantive regions of ventricular tissue diluting the effects of septal activation. The R wave amplitude is complex, yet ultimately shows a decrease in amplitude when adding stimuli. The seemingly anomalous large increase in S wave amplitude resembles RBBB morphology, as shown in the QRS complex signal (Fig. 4). Here, all three stimuli are confined to the left ventricular myocardium, and no stimuli are in the right ventricular myocardium, essentially simulating RBBB: in lead I QRS duration is greater than 120 ms (130 ms) and the slurred S wave is longer than 40 ms (50 ms).^25^ Note, the net negative deflection in lead III along with normal polarities in leads I and II indicate that the QRS axis is slightly shifted leftward.

Overall, the QRS complex feature metrics are sensitive to the number of seed stimuli, with the most sensitive metrics being QRS duration, and the R and S wave amplitudes. The trends in the metrics as the number of stimuli increase are different for each metric, and some are non-monotonic. In reality, representing activation in each IAR by one stimulus per region is an oversimplification. Thus, in the next section, the consequences of adding “regional” activation stimuli around the seed stimuli are analyzed, in terms of the effects on the QRS complex feature metrics.

### Sensitivity of QRS Complex Features to Number of Regional Stimuli Around the Four Seed Stimuli

**Fig. 5:**
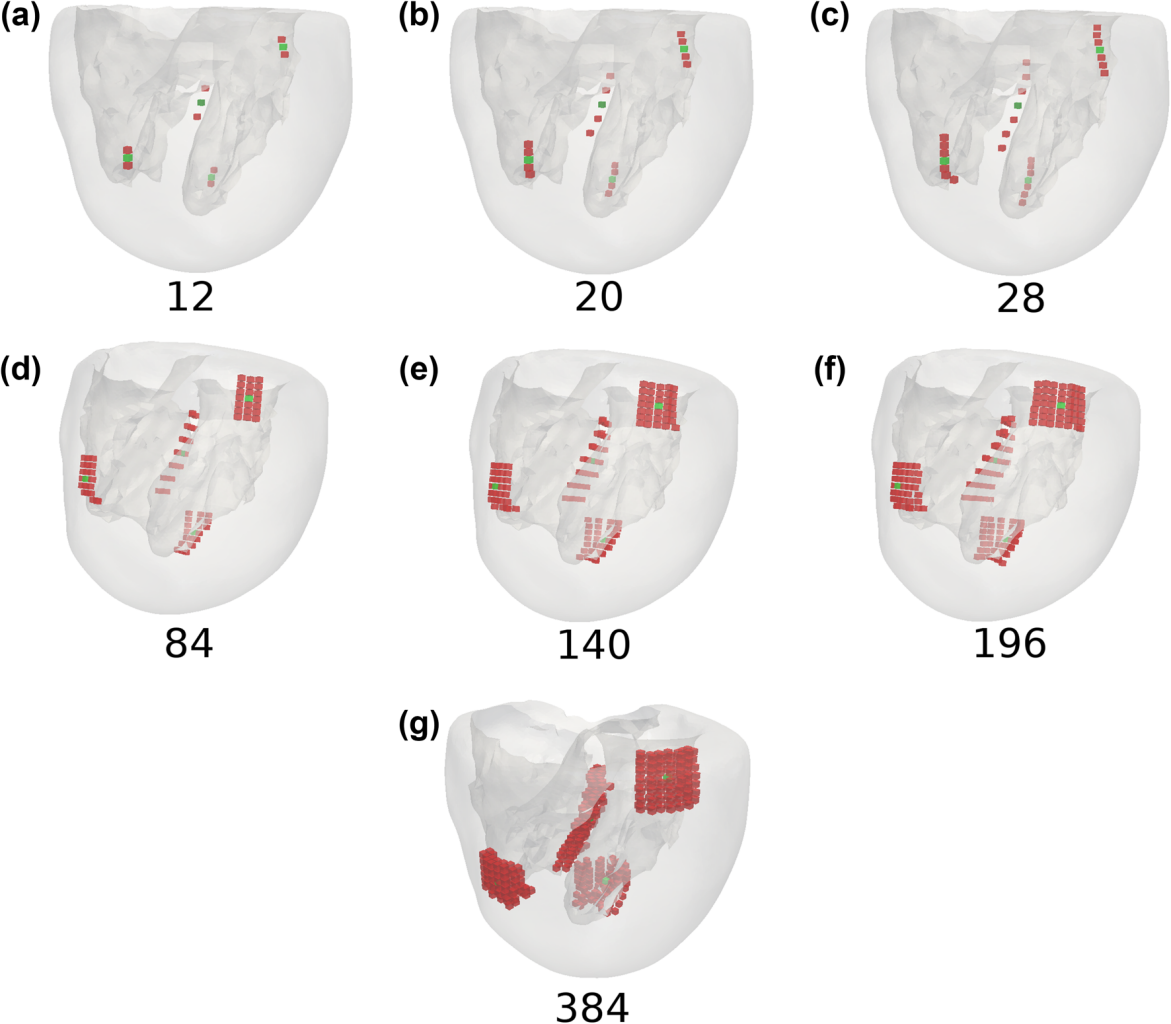
Sequential addition of regional stimuli around the seed stimuli in each IAR (refer to Figs. 1b and 1c for IARs). Configurations in (a–c) add stimuli in the apico-basal direction, (d–f) add stimuli in left-right/anterior-posterior directions, and (g) adds one layer of stimuli in the transmural direction. The perspective changes slightly between rows to enhance clarity as stimulus boxes are added in different directions. The total number of stimuli in each configuration are given below each panel.

Sensitivity of the QRS complex feature metrics to increasing number of regional stimuli in each IAR is analyzed. Regional stimuli are added layer by layer around each of the four seed stimuli, as shown in Fig. 5. Given a lack of experimental data on the number of PMJs, regional stimuli were added until the total number of stimuli in the ventricles was in the hundreds, on the order of previous modeling studies.^3^,^27^

**Fig. 6:**
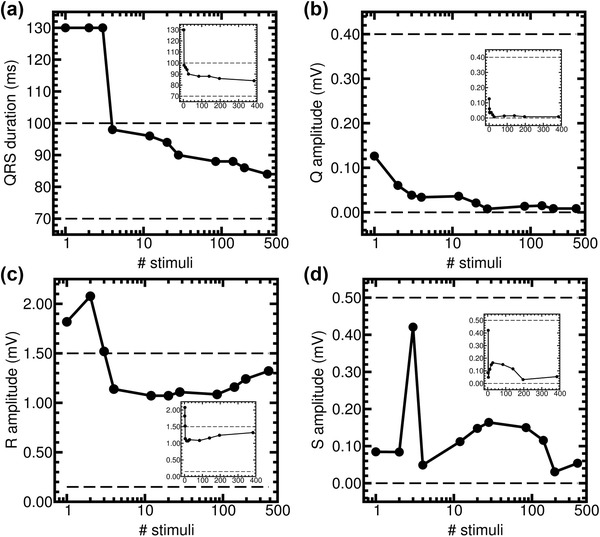
Values of QRS complex features as a function of the number of seed stimuli (4 or fewer stimuli) and regional stimuli (12 or more stimuli), up to a maximum of 384 total stimuli. Regional stimuli are progressively added symmetrically around the four initial seed stimuli (see Fig. 5). For each panel, the thin dashed lines indicate upper and lower physiological thresholds for the normal QRS complex (from Chou’s Electrocardiography In Clinical Practice^25^). The inset in each panel shows the same data using a linear x-axis to more intuitively visualize trends as the number of stimuli increase. Panels show the QRS duration measured in lead I (a), the Q wave amplitude measured in lead I (b), the R wave amplitude measured in lead I (c), and the S wave amplitude measured in lead II (d). Lead positions on torso are shown in Fig. 1a.

**Figure 7:**
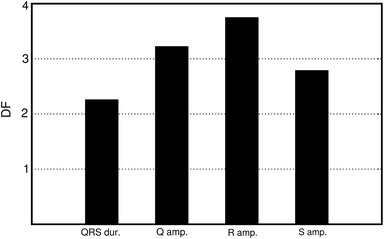
*DF* calculated for each QRS complex feature metric, using Eq. (2). *DF* is a ratio that measures the degree to which the QRS complex features are desensitized to the addition of regional stimuli. *DF* is larger than unity for all the feature metrics, and, thus, all metrics are more sensitive to seed stimuli than regional stimuli; the magnitude of *DF* indicates the degree to which a metric is more sensitive to seed stimuli.

Figure 6 reveals that QRS complex feature metrics are less sensitive to an increase in regional number of stimuli (local density) than the number of seed stimuli. This observation is now examined in detail using several different approaches. First, visual observation of the panels in Fig. 6 indicates that the changes in QRS complex features from addition of regional stimuli (greater than 4 stimuli) are not significant enough to bring any of the features in or out of normal physiological range. Compare this observation to the effects from adding seed stimuli (4 or less stimuli), which can bring metrics in and out of normal physiological range. Second, desensitization to adding regional stimuli is calculated *via* a desensitization factor (*DF*) reported in Fig. 7. *DF* is calculated by first dividing each of the four QRS complex metrics (i.e., QRS duration and Q/R/S wave amplitudes) into two categories, less than 4 (seed stimuli) or greater than 4 (addition of regional stimuli), and then finding the ratio of the statistical range of the metric in each category,

2 $$DF = \frac{{{\text{range(\{metric}}_{\text{N}} :1 \le N \le 4{\text{\})}}}}{{{\text{range(\{metric}}_{\text{N}} :4 \le N \le 384{\text{\})}}}},$$

where *metric*_N_ is the value of a particular QRS complex metric from using the configuration with *N* total number of stimuli (from Fig. 6). All *DF* values are greater than one, indicating the QRS complex is more sensitive to adding seed stimuli than regional stimuli. Still, to the degree that each metric *is* sensitive to adding *regional stimuli* relative to seed stimuli, the QRS duration is most sensitive (smallest *DF*), followed by the S, Q, and R wave amplitudes. These relative sensitivities indicate how useful adding regional stimuli may be in fitting different QRS complex features. For example, regional stimuli may be more important in fitting the QRS duration, and less important in fitting the R wave amplitude.

**Figure 8:**
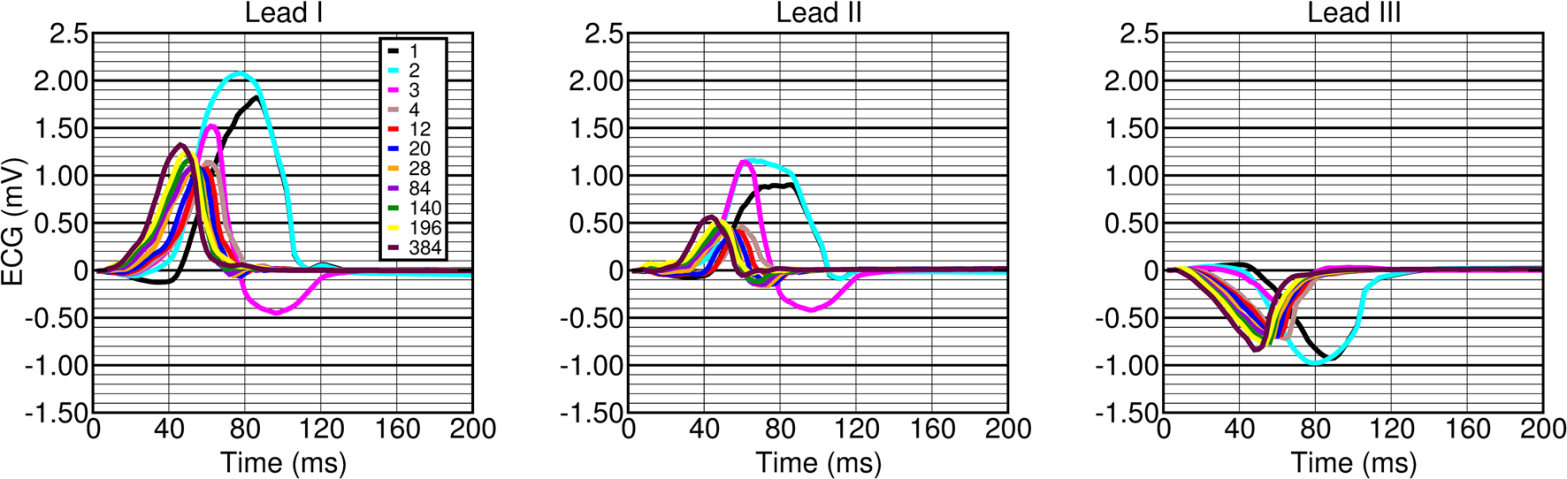
QRS complex of the ECG in leads I, II, and III for all eleven stimuli configurations in Figs. 2 and 5, corresponding to eleven feature metric data points in each panel of Fig. 6. Thick grid lines indicate 0.5 mV and 200 ms increments and thin grid lines indicate 0.1 mV and 40 ms increments, in accordance with the standard ECG. The ratio of x/y-axes is not in accordance with the standard ECG to increase clarity.

Third, the aforementioned desensitization to adding regional stimuli is also apparent from the simulated QRS complexes themselves. Figure 8 shows QRS complexes for all eleven stimuli configurations, corresponding to the eleven data points for each metric in Fig. 6 and the eleven stimuli configurations in Figs. 2 and 5. As seed stimuli are added (black, cyan, magenta, and light brown curves), there are visually significant changes in the curves. Adding regional stimuli to the seed stimuli (all other colored curves) also produce changes in the QRS complex, yet changes are less dramatic. Specifically, there are more subtle changes in R wave amplitude, QRS duration, and apparent initiation time of the QRS complex.

**Figure 9:**
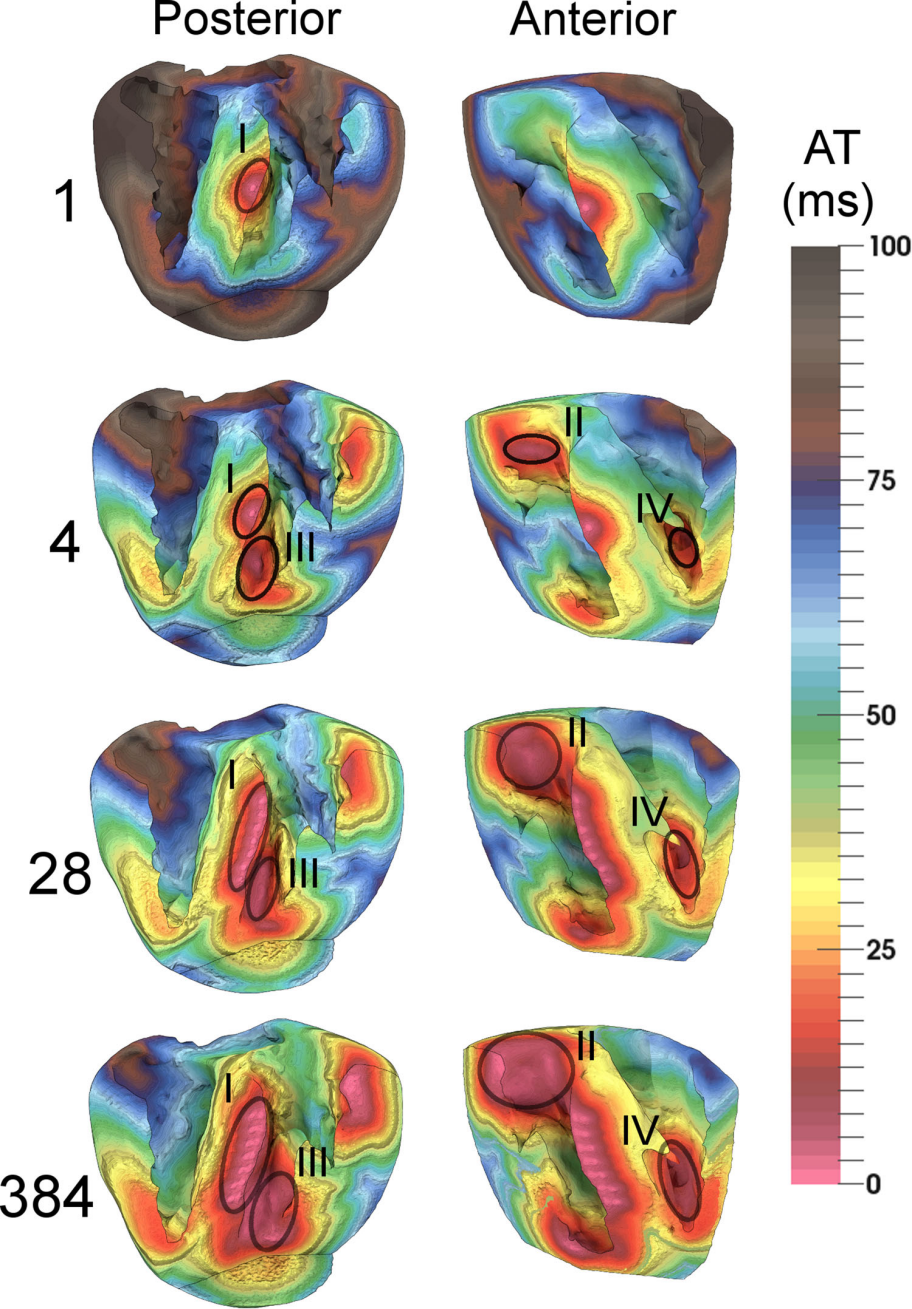
AT isochrones maps. For direct comparison, times of activation (in ms) are color-coded to match the color scheme of Durrer *et al.*,^9^ except that more detail is favored in this map by using more color contour divisions. Isochrone maps are shown for posterior and anterior halves for 1, 4, 28 and 384 stimuli configurations (see Figs. 2 and 5). Black circles show four IARs comparable to those of Durrer *et al.*: (I) the midseptum, (II) the anterior left ventricular wall near the base, (III) the posterior left ventricular wall in the paraseptal region approximately one-third the distance between the apex and base, and (IV) the right ventricular wall near the insertion of the anterior papillary muscle. A geometric smoothing filter was applied to the mesh without modification of color contour data or major geometrical features.

Fourth, the aforementioned desensitization to adding regional stimuli is also observable in the ventricular isochrone activation maps. Figure 9 shows the isochrone activation maps from four stimuli configurations: using just one seed stimulus, four seed stimuli, using a relatively small number of additional regional stimuli (28 stimuli total), and using a large number of regional stimuli (384 stimuli total). Certainly as expected, there is a large change in the AT map between using one and four seed stimuli, and using just one stimulus produces an AT that looks nothing like the experimental results of Durrer *et al.*^9^ Using four or more stimuli, the AT maps do change, but to a lesser extent. The change between using four and twenty-eight stimuli is much more pronounced than between using 28 and 384, a trend that is investigated more using QRS complex metrics in the next section. Using four or more stimuli, the pattern of activation qualitatively matches IARs seen in experiments by Durrer *et al.*^9^ (circles in Fig. 9): I) the midseptum, II) the anterior left ventricular wall near the base, III) the posterior left ventricular wall in the paraseptal region about one-third the distance between apex and base, and IV) the right ventricular wall near the insertion of the anterior papillary muscle.^9^ Specifically, in each IAR, there is at least a small volume of tissue that matches earliest ATs seen in Durrer *et al.*: 0–5 ms in the septum and left ventricle (black circles I–III in Fig. 9), and 5–10 ms in the right ventricle (black circle IV in Fig. 9).

**Figure 10:**
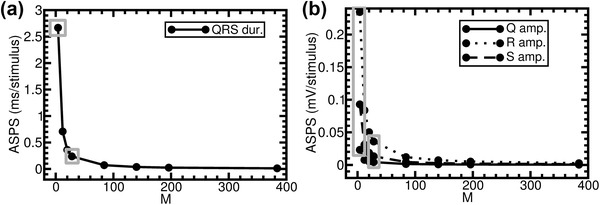
Average sensitivity per stimulus (*ASPS*, Eq. (3)) for QRS complex feature metrics as a function of maximum number of regional stimuli considered in calculating *ASPS* (*M* = 4, 12, 20, 28, 84, 140, 196, 384). Values of *ASPS* for QRS duration are shown in (a), and values of *ASPS* for wave amplitudes are shown in (b). Grey rectangles highlight metrics for *M* = 4 and *M* = 28.

Finally, in the observed desensitization, it is important to consider the application of patient-specific modeling, where fitting parameters in the model that do not have significant effect on the QRS complex features is undesirable. While QRS complex features are more sensitive to seed than regional stimuli, it is still prudent to examine the per-stimulus effect on the QRS complex features from adding regional stimuli. Figure 10 shows a calculation of diminishing returns as more and more regional stimuli are added, reported as average sensitivity per stimulus (*ASPS*),

3 $$ASPS = \frac{{{\text{avg}}\left[ {\left\{ {{\text{abs}}\left( {\Delta metric_{N} } \right) :1 \le N \le M} \right\}} \right]}}{M},\quad \left\{ {M = 4,12,20,28,84,140,196,384} \right\},$$

where ∆*metric*_*N*_ is the change in the particular QRS complex metric value (i.e., QRS duration and Q/R/S wave amplitudes) from using the configuration with *N* total number of stimuli vs. using the configuration with next smallest value of *N* (calculated from Fig. 6). *M* is the chosen maximum number of total stimuli considered in calculating a particular *ASPS* value. Thus, the *ASPS* for a particular value of *M* is interpreted as the average sensitivity contributed per stimulus for *N* ≤ *M*, divided by the maximum number of stimuli considered (*M*). The higher *ASPS* is, the more sensitive the QRS complex feature metric is to each stimulus (using *M* or less total stimuli). Figure 10 indicates that for all metrics, the average sensitivity per stimulus using at most four seed stimuli (*M* = 4, first grey square in both panels of Fig. 10) is higher than the sensitivity per stimulus for any number of additional regional stimuli. Thus, the QRS complex features are most sensitive to seed stimuli on a per-stimulus basis. Furthermore, all metrics for *M* > 4 show a per-stimulus change that is small: only a fraction of a standard ECG unit (0.1 mV by 40 ms). The 28 stimuli configuration (second grey rectangle in both panels of Fig. 10) is a reasonable ceiling on the number of stimuli needed for activation of a healthy heart for three reasons. First, for *M* > 28, the maximum per-stimulus change in QRS duration is less than 0.2% of 40 ms, and the maximum per-stimulus change in wave amplitudes is 10% of 0.1 mV. Second, from visual inspection, at *M* = 28 is where the slopes of *ASPS* for all metrics start leveling off, thus indicating that not much precision is gained by using more than 28 stimuli. Third, using 28 stimuli, Fig. 6 indicates that all metrics are well within normal physiological range with significate buffer zones between the metric value and non-physiological values. Ultimately, it is undesirable to make a model more complex than required by having more stimuli than necessary to reproduce patient-derived QRS complex features, and, therefore, 28 stimuli appears to be a reasonable upper bound from both precision and physiological considerations.

### Sensitivity of QRS Complex Features to Number Versus Topographical Extent of Stimuli

**Figure 11:**
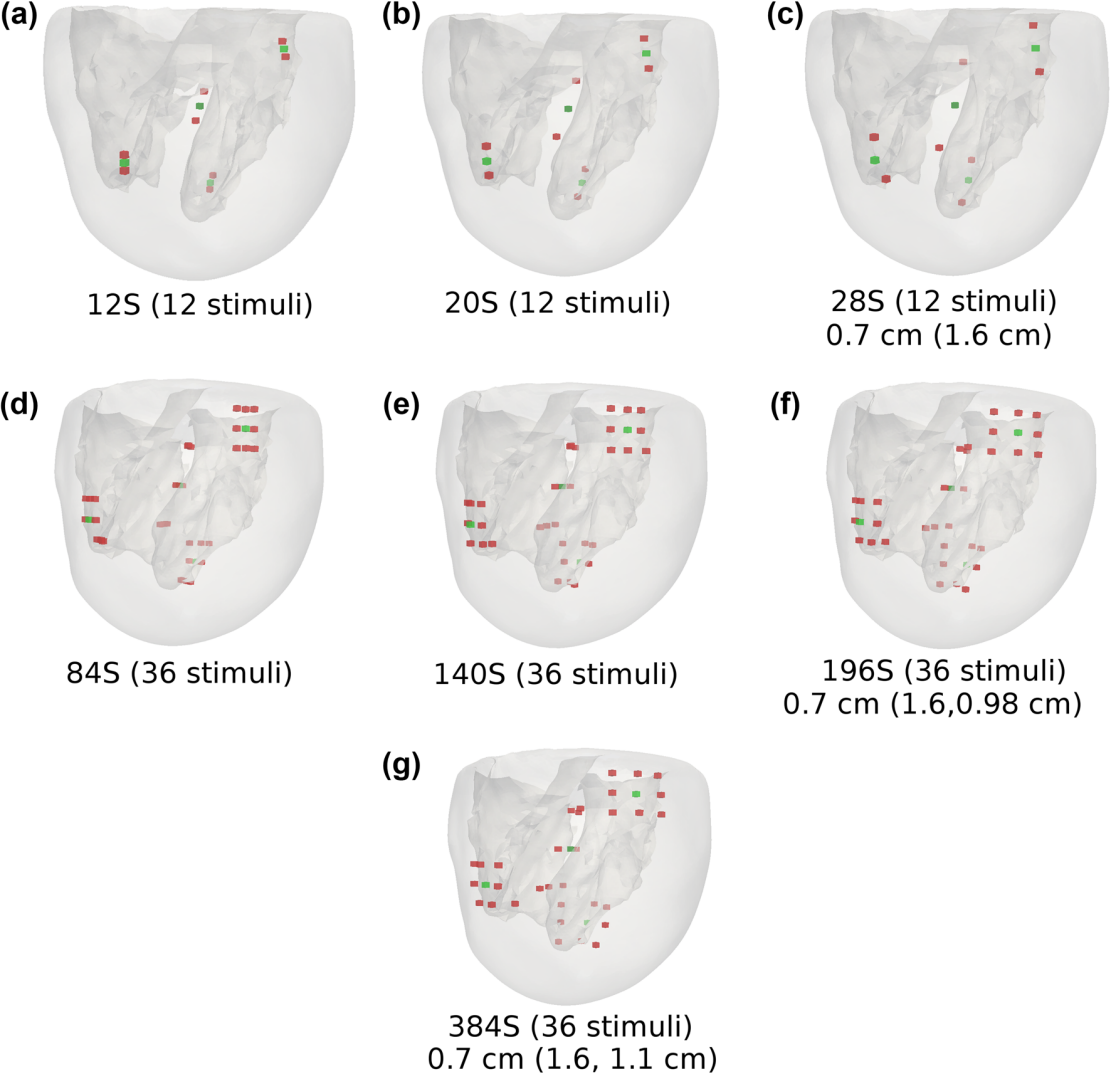
Sparse stimuli configurations for all dense configurations in Fig. 5. Sparse configurations (indicated by the appended “S”) capture the same topographical extent of stimuli in each IAR, but the number of stimuli bounded by the most peripheral stimuli are reduced. The perspective changes slightly between panels to enhance clarity. Configuration name, and total number of stimuli in each sparse configuration (in parentheses), are given below each panel. For maximum extension configurations, the regional inter-stimuli distance in regions LASW, LPIW, and RAIW (not in parenthesis) and in region S (in parenthesis, apico-basal direction and anterior-posterior direction) are given. Sparse configuration 12S (a) is exactly the same as dense configuration 12 in Fig. 5a.

**Figure 12:**
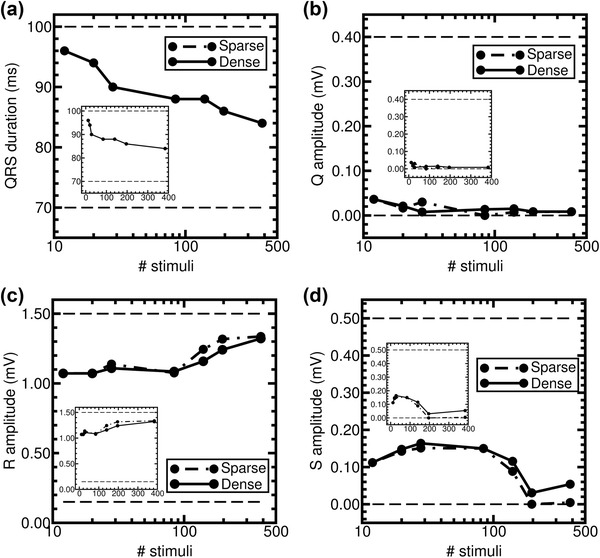
Comparing values of QRS complex feature metrics for sparse vs. dense stimuli configurations. Metrics shown for regional stimuli, i.e., between 12 and 384 total stimuli (# stimuli based on dense configurations). Metrics for dense configurations (Fig. 6) are shown as solid line, while metrics from sparse configurations (Fig. 11, “S” configurations) are shown as dashed-dotted line. All other descriptors are the same as in the caption for Fig. 6.

**Figure 13:**
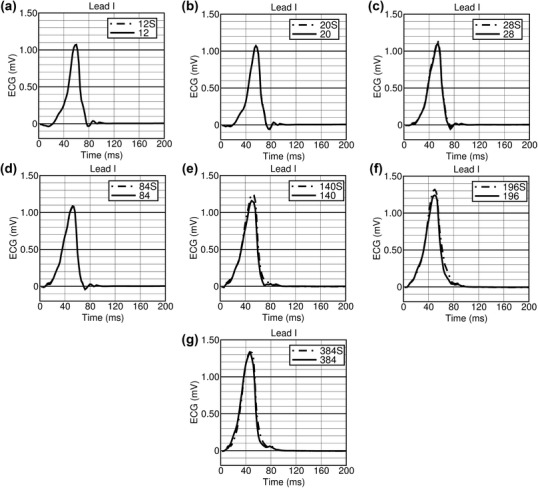
QRS complex of the ECG for sparse (Fig. 11) vs. dense (Fig. 5) stimuli configurations from lead I. Similar trends are seen in leads II and III, which are not included to enhance readability of the figure. For each panel, results from sparse configurations use a dash-dot line, while dense configurations use a solid line. Thick grid lines indicate 0.5 mV and 200 ms increments and thin grid lines indicate 0.1 mV and 40 ms increments, in accordance with the standard ECG. The x/y-axes ratio are not scaled to the standard ECG to increase clarity.

Adhering to the goal of minimizing the number of stimuli while maintaining precision and control over the QRS complex features (e.g., for model parameter fitting), the relative effect of number vs. topographical extent of stimuli is investigated. The topographical extent of stimuli is defined as the distance between seed stimulus and most peripheral regional stimuli, and in the previous analysis it is evident that as regional stimuli are added, this extent also changes. To separate number from topographical extent effects, each of the seven configurations in the previous analysis that add regional stimuli around the seed stimuli (Fig. 5) are considered. Sensitivity simulations are repeated by retaining the peripheral regional stimuli (and the seed stimulus) in each configuration in Fig. 5, but the number of regional stimuli bounded by the peripheral stimuli are reduced. For a visual reference, compare “dense” configurations in Fig. 5 to “sparse” configurations in Fig. 11. Figure 12 indicates that QRS complex features are very similar between dense and sparse configurations. While some differences appear significant, the scale of y-axis must be kept in mind; in fact, the maximum differences are 0 ms for QRS duration, 0.022 mV for Q wave amplitude, 0.086 mV for R wave amplitude, and 0.049 mV for S wave amplitude. These differences represent only fractions of the standard ECG precision, except for the difference in R wave, which is indeed very close to the 0.1 mV precision. Figure 13 shows how these small differences manifest in lead I of the QRS complex. Observing these complexes, if the topographical extent of stimuli is preserved, a nearly identical QRS complex is produced even when decreasing the number of stimuli by ten-fold (e.g., 384S sparse configuration of 36 stimuli and 384 dense configuration of 384 stimuli, Fig. 11g). Thus, overall, the number of regional stimuli has much less effect than the topographical extent of the stimuli. The previously found upper bound of 28 stimuli to simulate activation in a normal heart is reduced to only 12 stimuli if arranged appropriately (compare sparse configuration 28S in panel (c) of Figs. 11 and 13 to dense configuration 28 in panel (c) of Figs. 5 and 13).

### Bundle Branch Block (BBB) Simulations

**Figure 14:**
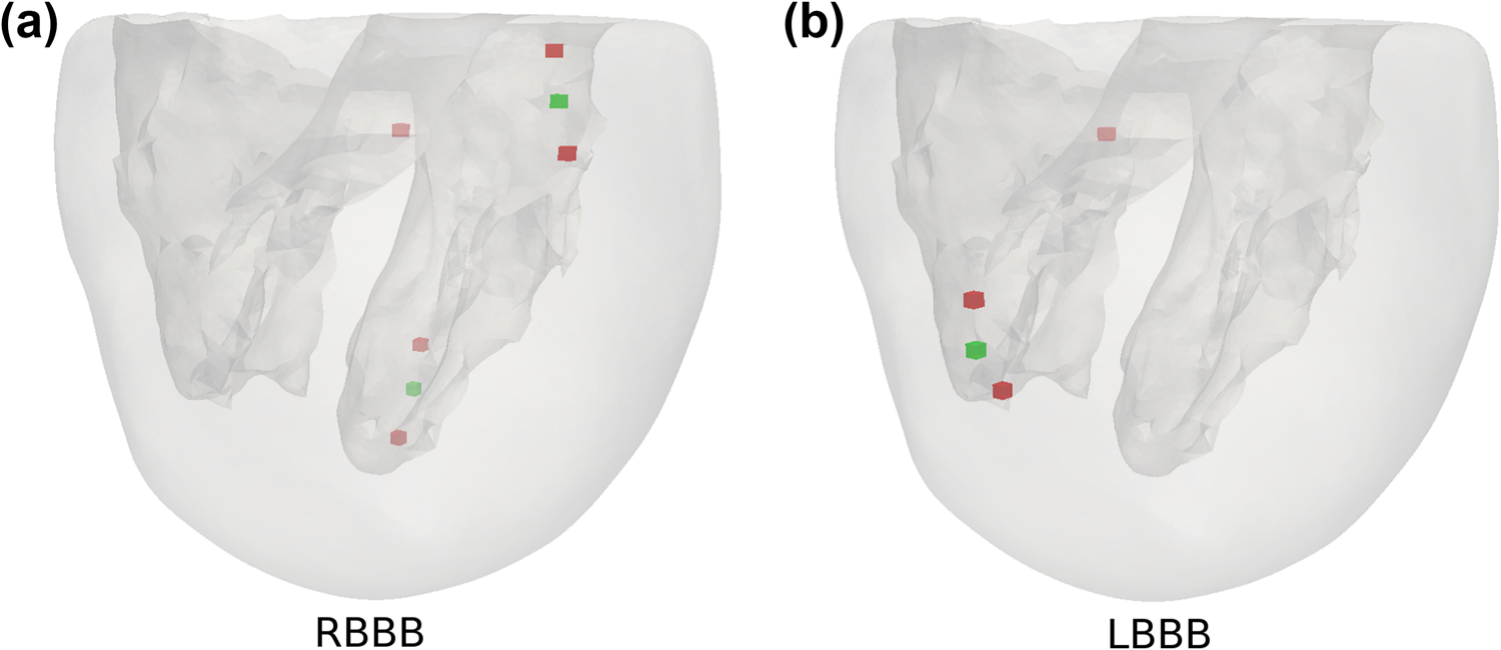
Stimuli configurations used for RBBB and LBBB. The stimuli and their locations within the ventricles are shown for RBBB (a) and LBBB (b). Both BBB stimuli configurations are derived from the 12-stimuli sparse configuration (Fig. 11c). Starting from the 12-stimuli sparse configuration, RBBB is simulated by removing stimuli in the right ventricle, and LBBB is simulated by removing stimuli in the left ventricle. For both BBBs, the basal, septal stimulus is positioned to emulate the physiological delay in the depolarization “wave” from fibrotic block in the infra-Hisian bundle of interest.

**Figure 15:**
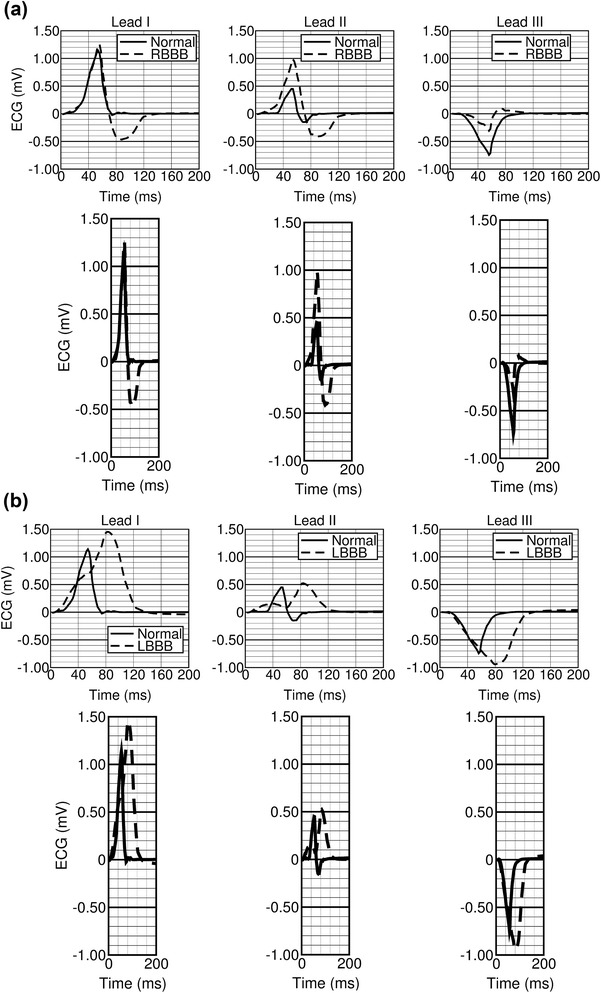
QRS complex of the ECG in leads I, II, and III for RBBB (a) and LBBB (b), refer to BBB stimuli configurations in Fig. 14. For both panels, the solid line is the “normal” QRS complex from using sparse 12-stimuli configuration (Figs. 11c and 13c), while the dashed line is the BBB QRS complex. Thick grid lines indicate 0.5 mV and 200 ms increments and thin grid lines indicate 0.1 mV and 40 ms increments, in accordance with the standard ECG. Top row in each panel uses *x*/*y*-axes ratio not in accordance with the standard ECG to increase clarity, while the bottom row does use the proper ratio where 0.1 mV and 40 ms intervals are equivalent Euclidean distances.

Having examined the sensitivities in the context of a normal QRS complex, and having found a reasonable minimal 12-stimuli protocol for normal activation, simulating a diseased state is now examined. Both BBB stimuli configurations are derived from the 12-stimuli sparse configuration (Fig. 11c), and are shown in Fig. 14. QRS complexes from simulations of RBBB and LBBB are shown in Fig. 15. Figure 15a exhibits signature QRS complex features of RBBB, including a long, slurred S wave in lead I with duration of 40 ms or greater (64 ms), as well as a total QRS duration of 120 ms or greater (140 ms).^25^ Interestingly, these same features are present when using the 3-stimuli configuration (Fig. 4), which is another example of the QRS complex being more sensitive to location than number of stimuli. Figure 15b exhibits the signature features of LBBB, including a total QRS duration of 120 ms or greater (132 ms) and notching in the upstroke of the R wave in leads I and II.^33^ Thus, the 12-stimuli sparse protocol is able to simulate the major features of healthy ventricular activation in the QRS complex, and simple alterations to this protocol yield major features of BBB.

### Sensitivity of QRS Complex Features to Heterogeneous Versus Homogeneous Material Torso Models

**Figure 16:**
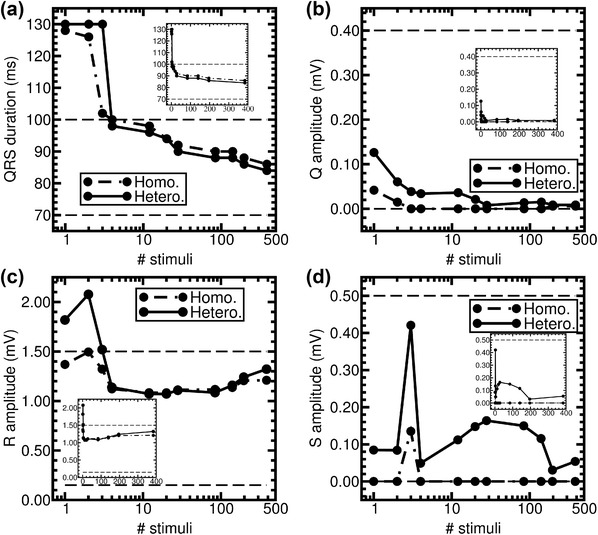
Values of QRS complex feature metrics as a function of the number of stimuli, using heterogeneous (data from Fig. 6) vs. homogeneous material torso composition. For each panel, the dashed-dotted lines are the signals generated using homogeneous torso, and solid lines are the signals generated using heterogeneous torso. The S wave was not detectable for the homogeneous material torso except when using 3 stimuli. All other descriptors are the same as in the caption for Fig. 6.

**Figure 17:**
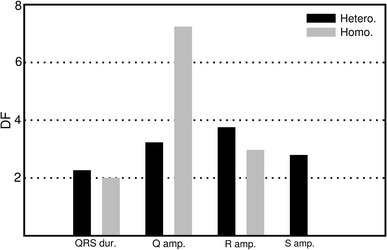
*DF* calculated for each QRS complex feature metric, using Eq. (2), for heterogeneous (see Fig. 7) and homogeneous torso material model. *DF* is a ratio that measures the degree to which the QRS complex features are desensitized to the addition of regional stimuli, relative to the addition of seed stimuli. *DF* is larger than unity for all the feature metrics, and, thus, all metrics are more sensitive to seed than regional stimuli; the magnitude of *DF* indicates the degree to which the metric is more sensitive to seed stimuli. For homogeneous case, the S wave is not detectable for regional stimuli configurations (using more than 4 stimuli, see Fig. 5), and, thus, the *DF* does not exist.

In a departure from examining only the relationship between number and location of stimuli in the ventricles, the effects of torso material composition are now investigated. Some models in the cardiac electrophysiology community do not include a torso model, and some of those that do include it do not model heterogeneous material composition. Thus, as a first step in assessing the effect that the heterogeneous tissue materials have on the sensitivity results, analysis of the QRS complex features is repeated using a homogeneous tissue material torso model. In Fig. 16, for all features except S wave amplitude, trends similar to the heterogeneous case are observed in the homogeneous case, except the values of the features have smaller magnitude. The S wave amplitude for homogeneous torso material is qualitatively different from the heterogeneous case: the S wave is absent except for the *N* = 3 configuration. Thus, the variation in S wave amplitude as the number of regional stimuli changes appears to be linked to torso composition. *DF* is generally lower (Fig. 17) for all homogeneous torso metrics, except the Q wave amplitude, where the sensitivity to seed stimuli is small enough that it yields a larger *DF* than the heterogeneous case. Overall, the *DF* values for the homogeneous case are still greater than one; thus, the QRS complex features are still more sensitive to presence/absence of seed stimuli than the number of regional stimuli.

## Discussion

QRS complex sensitivity simulations were performed using a biventricular and torso model by varying the number and location of current injection stimuli (i.e., PMJs) in the biventricular model. Locations of stimuli were constrained to the approximate volume of tissue corresponding to the IARs identified in Durrer *et al.*^9^ Simulations revealed relative sensitivity differences between altering number vs. location of stimuli. Overall, results suggest that patient-specific modeling of PMJs should focus parameter fitting on the location of just a few stimuli in each IAR, rather than parameterizing the number of stimuli. This trend is observed in several of the results. First, the *DF* (Figs. 7 and 17) quantifies the decrease in sensitivity observed in the QRS complex feature metrics (Figs. 6 and 16) to increasing number of stimuli at the regional scale. The *DF* values indicate that all QRS complex features are at least two-fold more sensitive to adding up to only 4 seed stimuli than to adding up to 380 regional stimuli, regardless of torso composition. In fact, for modeling healthy hearts, the QRS complex features attain physiological values when one stimulus per IAR is used. Thus, having at least one seed stimulus in each IAR may be thought of as a prerequisite for any further parameter fitting of stimuli that may follow. Second, it is evident that location plays a greater role in simulating BBB diseased states: RBBB simulated using only 3 seed stimuli (Fig. 4) produced similar QRS complex features as using 7 stimuli (Fig. 15a). Third, to the degree that the QRS complex features are sensitive to regional stimuli, it is the topographical extent of the stimuli rather than number of stimuli that make the difference (Figs. 12 and 13). In fact, for a given topographical extent of stimuli, the QRS complex shows virtually no sensitivity to the number of stimuli situated between the peripheral stimuli even when total number of stimuli is decreased by more than an order of magnitude (384 to 36 stimuli in Fig. 13g). Thus, for reproducing QRS complexes, it appears sufficient to vary regional topographical extent using no more than 36 stimuli distributed evenly amongst the four IARs (Figs. 11, 12, and 13). Understanding the maximum number of stimuli needed is important in patient-specific modeling, as reducing number of stimuli decreases the parameter space that must be explored, which increases efficiency.

As mentioned above, the QRS complex features are less sensitive to regional stimuli than to seed stimuli, and within the context of patient-specific modeling, regional stimuli may be viewed as a “fine tuning” step in parameter fitting. This fine tuning may prove more effective for features with lower *DF* values, namely, QRS duration and S wave amplitude. Stated slightly differently, changing the topographical extent of regional stimuli may have a greater effect when trying to model QRS duration (especially smaller durations, Figs. 6 and 16) and S wave amplitude (e.g., RBBB, Fig. 6d) accurately. Given that the QRS duration is affected by many diseases, the locations of regional stimuli may be an important consideration in model fitting. Certainly, the importance of altering locations of regional stimuli was observed in simulating RBBB and LBBB, where the septal stimulus close to the base needed to be translated by small amounts to produce appropriate BBB QRS complex features (Figs. 14 and 15). In contrast, there may be less added value in altering locations of regional stimuli when the focus of fitting is on Q wave amplitude (e.g., myocardial infarction) and R wave amplitude (e.g., hypertrophy), given their high *DF* values (Figs. 7 and 17). Also, torso material composition appears to be another factor that determines how much the topographical extent of regional stimuli affects QRS complex feature metrics (Fig. 16). The S wave amplitude appears to be exceptionally sensitive to torso material composition, as it is absent for many stimuli configurations in the homogeneous torso material model. Thus, for certain applications where S wave amplitude fitting is critical, it may be worthwhile to incorporate heterogeneous materials in the torso model. The *DF* for the Q wave amplitude is even higher for the homogeneous torso material, which means adding regional stimuli is likely to have even less effect on the Q wave amplitude than for the heterogeneous torso material model. Ideally, fitting parameters to patient-derived QRS complexes will involve matching all QRS complex features, but understanding the sensitivity of individual features may be beneficial when the focus is on a diseased state that manifests as a particular feature.

Using regional stimuli for fine tuning may also be viewed from a precision argument: how much precision does one need to reproduce faithfully patient-derived QRS complex features? While the precision needed and focus on particular features will vary by application in patient-specific modeling, results indicate that it is sufficient to use 12 stimuli (i.e., 28 stimuli dense configuration in Fig. 10) aligned in the apico-basal direction with regional inter-stimulus distance of no more than 0.7 cm (1.6 cm in septum). Using more than 12 stimuli, and increasing the topographical extent in directions other than apico-basal, produce only a per-stimulus change in QRS duration of less than 0.08 ms, and in wave amplitude of 0.01 mV or less (Fig. 10). Obtaining these kinds of precisions is likely not needed in terms of disease diagnosis, and exceed standard ECG precision. It is also worth noting that QRS complex features appear more sensitive to regional stimuli being shifted in the apico-basal direction than in other directions, which may provide guidance in constraining location of stimuli in parameter fitting procedures. Overall, altering the location of 12 stimuli (4 seed, 8 regional) arranged in the apico-basal direction appears to be a reasonable compromise between ability to manipulate QRS complex features, and having drastically more stimuli than needed for fitting precision.

Our results both contrast and corroborate results from previous modeling studies that examine the sensitivity of the QRS complex to PMJ parameters. The findings of Simelius *et al.*^22^ did emphasize the importance of location of stimulus sites; yet, these authors attributed their importance primarily to producing physiological AT maps on the heart rather than the QRS complex. They found that the QRS complex is primarily affected by carefully balancing stimuli firing times in the left and right ventricles. While our work does not explore sensitivity to stimuli firing times, it does show that the location of stimuli (topographical extent) affects the QRS complex, in addition to the AT maps. In fact, even in absence of the more sophisticated stimuli firing timings used in other studies, we were able to reproduce some of the same general phenomena in these studies. Specifically, we simulated signature BBB QRS complex features by removing specific stimuli (Fig. 15), altered magnitudes of QRS complex features *via* torso material manipulation (Fig. 16), and altered the S wave *via* changes in apico-basal location of stimuli (Fig. 12d).^7^ Regarding the S wave, studies by Potse *et al.*^19^ found it difficult to match simulated-to-measured S wave amplitudes. Our results combined with those of Cardone-Noott *et al.*^7^ suggest employing apico-basal location changes of stimuli (Fig. 12d), as well as alteration of torso composition (Fig. 16d), for control of S wave amplitude. Overall, despite these trends, the relationships between number, location, and activation timing of stimuli deserves further investigation in future studies.

Other studies that model the full Purkinje network tend to focus on obtaining high density of the network and the PMJs.^3^,^8^ Costabal *et al.*^8^ used special techniques to achieve smaller maximum distance between branches in generating the network over irregular geometric domains, which are ubiquitous in the heart. In contrast, our work suggests that emphasis on density and number of PMJs in the network may be less important for reproducing QRS complex features than strategic, physiologic placement of a limited number of PMJs.

Still, the added sophistication of a dense, anatomically accurate Purkinje network representation may be required for some modeling applications, even if not for all. For example, it is clearly advantageous to model a full Purkinje network if network topology or Purkinje cell function is the focus of investigation. Furthermore, studies such as those by Behradfar *et al.* have found that the number of junctions in a Purkinje network model plays a role in simulating reentry during arrhythmia.^3^ Their work was performed using a rabbit heart model, but when geometry is scaled to human size, they varied the number of PMJs between 1000 and 5000. Thus, while in our study relatively little added value is found beyond using 12 stimuli (the 28 stimuli dense configuration in Fig. 10) to simulate a healthy heart, there may be value in having more stimuli to simulate diseased hearts. Nayak *et al.* also found that increasing number of PMJs resulted in little-to-no spiral-wave breakup activity.^14^ Thus, future directions should include a sensitivity analysis of a wide range of ventricular disorders. To do so, our model may need to be altered in significant ways, given the Purkinje network fibers can act as a current sink during arrhythmias.^4^,^13^,^34^ This effect is not currently captured in the model and is beyond the scope of the current study, but would be an important addition in future studies.

Another outcome of the sensitivity analysis is a simple 12-stimuli configuration for modeling ventricular activation without the full Purkinje network. Such a simple protocol may be useful for studies in the community where it is desirable to model activation, but with a primary focus on diseases and phenomena downstream of initial activation, such as defects in ionic channel activities of myocytes and their impact on whole-organ ventricular dynamics. Still, even at the stage of initial activation, the 12-stimuli configuration has some relevance in modeling disorders, as shown for simulating QRS complex features of RBBB and LBBB (Figs. 14 and 15).

In the next phase of this work, the analysis will be expanded to include a more comprehensive examination of sensitivity in other disease states, and additional parameters of activation beyond number and location of stimuli will be examined. Furthermore, given that these results were derived from a single biventricular/torso model, the analysis will be performed in a variety of image-based heart and torso sets to test the repeatability and universality of the sensitivity results across users and geometries. Factors such as heart position and orientation will be evaluated in terms of how they affect sensitivity of the QRS complex features to stimuli parameters. Such studies could be compared to the work of Nguyên *et al.*^16^ Their research varied heart position and orientation, and examined effects on the QRS complex, but they did not alter initial ventricular activation parameters.

## Conclusion

In summary, this sensitivity analysis models PMJs as current injection stimuli in one biventricular/torso model, and investigates the sensitivity of simulated QRS complex features to number and location of these stimuli. We found that the QRS complex features are most sensitive to the locations of a few well-placed stimuli rather than to the sheer number of stimuli. The presence or absence of just one seed stimulus per IAR has significant effects on the QRS complex features, and to the extent that the regional stimuli affect QRS complex features, the topographical extent of relatively few stimuli (between 12 and 36) dominates QRS complex features. The QRS complex features are more sensitive to seed stimuli, but regional stimuli changes may offer “fine tuning” of these features to match patient-derived data. The stimulus configuration with 12 total stimuli oriented in an apico-basal direction and regional inter-stimuli distance of 0.7 cm (1.6 cm in septum) produced QRS complex features of a healthy heart with reasonable precision. This 12-stimuli configuration also served as a good basis for simulating BBB, yet it is undeniable that there are certain disease states and application spaces where a dense, full Purkinje network is useful. Ultimately, this work establishes a new approach in describing the sensitivity of QRS complex features for patient-specific modeling of activation. The QRS complex of the ECG is a noninvasive, standardized measurement of electrical activity in the ventricles, and thus is an attractive tool for model parameter fitting. Our results should be viewed within the context of our model limitations (current-injection stimuli model of PMJs), as well as viewed as a catalyst for future explorations of QRS complex sensitivities to numerous other PMJ parameters. Still, the results provided herein should aid in effectively decreasing the sheer number of parameters to be considered in fitting studies, and illuminate which parameters should be given the greatest weight, i.e., location over number. By providing knowledge that may increase the efficiency of fitting model parameters to clinically derived QRS complexes, this work represents another step towards the realization of patient-specific medicine. As the horizon of patient-specific medicine approaches, it brings with it the promise of lower risk and more effective treatment.

## References

[1] Aguado-Sierra J, Krishnamurthy A, Villongco C, Chuang J, Howard E, Gonzales MJ (2011). Patient-specific modeling of dyssynchronous heart failure: a case study. Prog. Biophys. Mol. Biol..

[2] Bayer JD, Blake RC, Plank G, Trayanova NA (2012). A novel rule-based algorithm for assigning myocardial fiber orientation to computational heart models. Ann. Biomed. Eng..

[3] Behradfar E, Nygren A, Vigmond EJ (2014). The role of Purkinje-myocardial coupling during ventricular arrhythmia: a modeling study. PLoS ONE.

[4] Berenfeld O, Jalife J (1998). Purkinje-muscle reentry as a mechanism of polymorphic ventricular arrhythmias in a 3-dimensional model of the ventricles. Circ. Res..

[5] Bishop MJ, Plank G, Burton RAB, Schneider JE, Gavaghan DJ, Grau V (2010). Development of an anatomically detailed MRI-derived rabbit ventricular model and assessment of its impact on simulations of electrophysiological function. Am. J. Physiol. Heart Circ. Physiol..

[6] Bordas R, Gillow K, Lou Q, Efimov IR, Gavaghan D, Kohl P (2011). Rabbit-specific ventricular model of cardiac electrophysiological function including specialized conduction system. Prog. Biophys. Mol. Biol..

[7] Cardone-Noott L, Bueno-Orovio A, Minchole A, Zemzemi N, Rodriguez B (2016). Human ventricular activation sequence and the simulation of the electrocardiographic QRS complex and its variability in healthy and intraventricular block conditions. Europace..

[8] Costabal FS, Hurtado DE, Kuhl E (2016). Generating Purkinje networks in the human heart. J. Biomech..

[9] Durrer D, Vandam RT, Freud GE, Janse MJ, Meijler FL, Arzbaecher RC (1970). Total excitation of the isolated human heart. Circulation.

[10] Krishnamurthy A, Villongco CT, Chuang J, Frank LR, Nigam V, Belezzuoli E (2013). Patient-specific models of cardiac biomechanics. J. Comput. Phys..

[11] Liu BR, Cherry EM (2015). Image-based structural modeling of the cardiac Purkinje network. Biomed. Res. Int..

[12] Mirin, A. A., D. F. Richards, J. N. Glosli, E. W. Draeger, B. Chan, J. L. Fattebert, et al. Toward real-time modeling of human heart ventricles at cellular resolution: simulation of drug-induced arrhythmias. In *Proceedings of the International Conference on High Performance Computing, Networking, Storage and Analysis*. Washington, DC: IEEE Computer Society Press, 2012.

[13] Monserrat M, Saiz J (1999). Reentry based on the development of early after depolarizations in a Purkinje-ventricular muscle ring model. Comput. Cardiol..

[14] Nayak AR, Panfilov AV, Pandit R (2017). Spiral-wave dynamics in a mathematical model of human ventricular tissue with myocytes and Purkinje fibers. Phys. Rev. E.

[15] Neal ML, Kerckhoffs R (2010). Current progress in patient-specific modeling. Brief. Bioinform..

[16] Nguyên UC, Potse M, Regoli F, Caputo ML, Conte G, Murzilli R (2015). An in-silico analysis of the effect of heart position and orientation on the ECG morphology and vectorcardiogram parameters in patients with heart failure and intraventricular conduction defects. J. Electrocardiol..

[17] Palamara S, Vergara C, Faggiano E, Nobile F (2015). An effective algorithm for the generation of patient-specific Purkinje networks in computational electrocardiology. J. Comput. Phys..

[18] Pan J, Tompkins WJ (1985). A real-time QRS detection algorithm. IEEE Trans. Bio-Med. Eng..

[19] Potse M, Krause D, Kroon W, Murzilli R, Muzzarelli S, Regoli FO (2014). Patient-specific modelling of cardiac electrophysiology in heart-failure patients. Europace..

[20] Richards DF, Glosli JN, Draeger EW, Mirin AA, Chan B, Fattebert JL (2013). Towards real-time simulation of cardiac electrophysiology in a human heart at high resolution. Comput. Methods Biomech..

[21] Scientific Computing and Imaging Institute. “Seg3D” Volumetric Image Segmentation and Visualization. Scientific Computing and Imaging Institute (SCI), 2015. http://www.seg3d.org/.

[22] Simelius K, Nenonen J, Horáček M (2001). Modeling cardiac ventricular activation. Int. J. Bioelectromagn..

[23] Stephenson RS, Atkinson A, Kottas P, Perde F, Jafarzadeh F, Bateman M (2017). High resolution 3-dimensional imaging of the human cardiac conduction system from microanatomy to mathematical modeling. Sci. Rep..

[24] Stephenson RS, Boyett MR, Hart G, Nikolaidou T, Cai X, Corno AF (2012). Contrast enhanced micro-computed tomography resolves the 3-dimensional morphology of the cardiac conduction system in mammalian hearts. PLoS ONE.

[25] Surawicz B, Knilans TK (2001). Chou’s Electrocardiography in Clinical Practice.

[26] ten Tusscher KHWJ, Panfilov AV (2006). Alternans and spiral breakup in a human ventricular tissue model. Am. J. Physiol. Heart Circ..

[27] Ten Tusscher KHWJ, Panfilov AV (2008). Modelling of the ventricular conduction system. Prog. Biophys. Mol. Biol..

[28] Trayanova NA (2011). Whole-heart modeling applications to cardiac electrophysiology and electromechanics. Circ. Res..

[29] U. S. National Library of Medicine. Visible Human Project®. National Institutes of Health, U.S. Department of Health & Human Services, 2015. https://www.nlm.nih.gov/research/visible/visible_human.html. Accessed 1 Feb 2017.

[30] Vadakkumpadan F, Gurev V, Constantino J, Arevalo H, Trayanova N, Kerckhoffs RCP (2010). Modeling of whole-heart electrophysiology and mechanics: toward patient-specific simulations. Patient-Specific Modeling of the Cardiovascular System: Technology-Driven Personalized Medicine.

[31] Vergara C, Palamara S, Catanzariti D, Nobile F, Faggiano E, Pangrazzi C (2014). Patient-specific generation of the Purkinje network driven by clinical measurements of a normal propagation. Med. Biol. Eng. Comput..

[32] Vigmond E, Vadakkumpadan F, Gurev V, Arevalo H, Deo M, Plank G (2009). Towards predictive modelling of the electrophysiology of the heart. Exp. Physiol..

[33] Wagner GS (2001). Marriott’s Practical Electrocardiography.

[34] Walter PF, Pollak S (1984). Rapid ventricular-tachycardia due to his-Purkinje reentry. PACE.

